# Supplementary material for: Efficient spin current source using a half-Heusler alloy topological semimetal with back end of line compatibility
Source: Sci Rep. 2022 Feb 14;12:2426. doi: 10.1038/s41598-022-06325-1 (PMC8844406; doi:10.1038/s41598-022-06325-1)
Supplement: Supplementary file 1 — Supplementary Information. [file 41598_2022_6325_MOESM1_ESM.pdf]

Supplementary Information

Efficient spin current source using a half-Heusler alloy topological semimetal with Back End of Line compatibility

Takanori Shirokura[1], Tuo Fan[1], Nguyen Huynh Duy Khang[1,2], Tsuyoshi Kondo[3], and Pham Nam Hai[1,4]*

[1] *Department of Electrical and Electronic Engineering, Tokyo Institute of Technology, Meguro, Tokyo 152-8550, Japan*

[2] *Department of Physics, Ho Chi Minh City University of Education, Ho Chi Minh City 738242, Vietnam*

[3] *Device Technology R&D Center, Institute of Memory Technology R&D, Kioxia Corporation, Kawasaki, Kanagawa 212-8582, Japan*

[4] *Center for Spintronics Research Network (CSRN), The University of Tokyo, Bunkyo, Tokyo 113-8656, Japan*

*Corresponding author: pham.n.ab@m.titech.ac.jp

1. Surface roughness analysis for YPtBi by X-ray reflectivity measurement

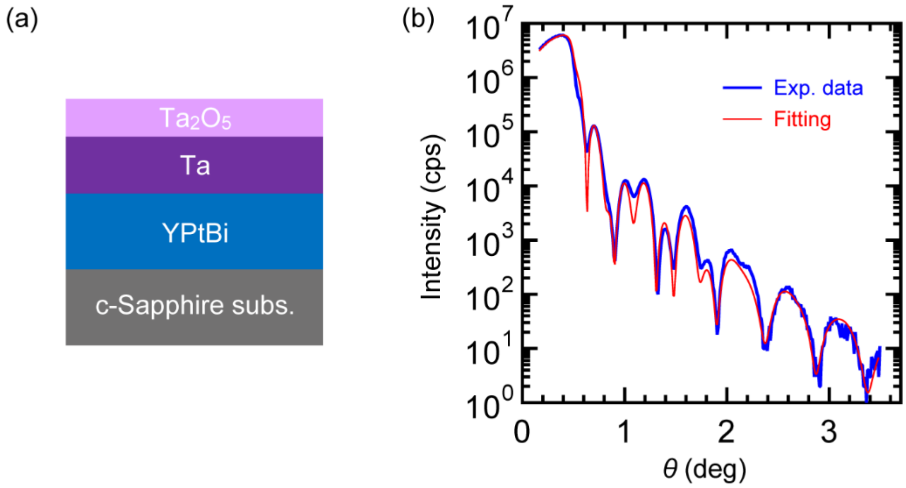

Figure S1. (a) Model of the YPtBi stack used for fitting of an XRR spectrum. (b) XRR spectrum for the YPtBi stack, where the blue and red solid lines are the experimental and fitting results, respectively.

We employed the X-ray reflectivity (XRR) method to evaluate the surface roughness of YPtBi with a Ta cap layer, which is necessary for protection of YPtBi from surface oxidization that makes difficult to evaluate the surface roughness of YPtBi by conventional surface analysis method such as the atomic force microscopy. For XRR measurement, we deposited ~10 nm-thick YPtBi with a 10 nm-thick Ta capping layer on a c-sapphire substrate. Because Ta is partly oxidized after exposure to the atmosphere, we employed a structure of Ta_2O_5/Ta/YPtBi/c-Sapphire for fitting, as shown in Fig. S1(a). Figure S1(b) shows a XRR spectrum of this stack, where the blue and red solid lines are the experimental and fitting results, respectively. The fitting is in good agreement with the experimental result. From this fitting, we obtained the YPtBi surface roughness of 2.4 Å, which is 3 times smaller than the surface roughness of BiSb topological insulator. This atomically flat surface of YPtBi is promising for application to spintronic devices such as MRAM with strong PMA.

2. Low-field second harmonic measurement for sample A

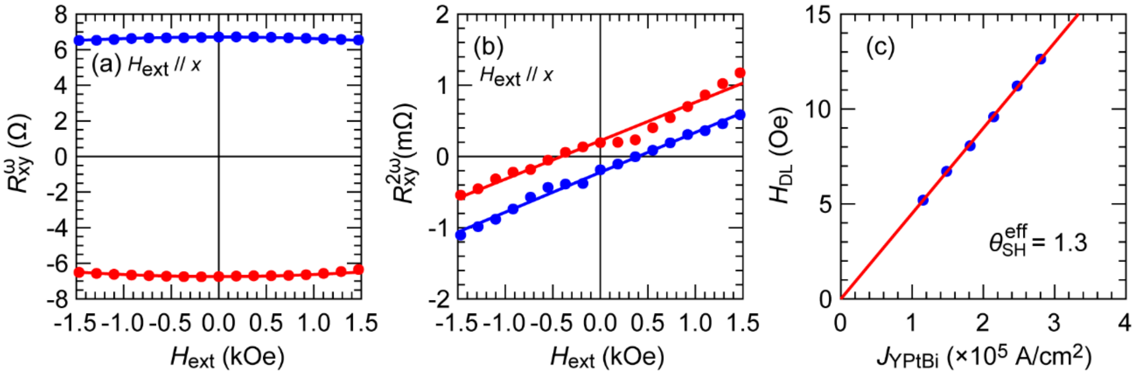

Figure S2. (a) First harmonic Hall resistance and (b) Second harmonic Hall resistance as a function of the external magnetic field applied along the *x*-direction for sample A with an applied alternating current of 1.4 mA. Here, dots and solid lines are experimental results and eye-guides, respectively. Blue (red) correspond to the up (down) direction of the magnetization. (c) Antidamping-like field as a function of the current density in the YPtBi layer.

Given that the thermal contribution is negligible in sample A, we also evaluated $\theta_{\mathrm{SH}}^{\mathrm{eff}}$ of sample A by using the low-field second harmonic technique to double check the large $\theta_{\mathrm{SH}}^{\mathrm{eff}}$ of 1.3 estimated by the high-field second harmonic technique. Figure S2(a) and S2(b) show the first and second harmonic Hall resistance R_{xy}^{ω} and $R_{\mathrm{xy}}^{2\omega}$ as a function of H_{ext}, respectively. Here, an alternating current of 1.4 mA at 259.68 Hz was applied. At low fields, R_{xy}^{ω} and $R_{\mathrm{xy}}^{2\omega}$ show quadratic and linear dependence of H_{ext}. We then evaluated H_{DL} by using following equation,

$$H_{\mathrm{DL}} = -2\frac{\partial R_{\mathrm{xy}}^{2\omega}/\partial H_{\mathrm{ext}}}{\partial^2 R_{\mathrm{xy}}^{\omega}/\partial H_{\mathrm{ext}}^2} \quad \text{(S1)}$$

Here, we neglect the field-like field H_{FL} term because this term was undetectable in the high-field second harmonic measurements with large H_{ext}. Figure S2(c) shows H_{DL} as a function of J_{YPtBi}. The value of $\theta_{\mathrm{SH}}^{\mathrm{eff}}$ calculated from the slope of H_{DL} / J_{YPtBi} is 1.3, which is consistent with that estimated by the high-field second harmonic technique in the main text. Therefore, we confirmed that sample A has large $\theta_{\mathrm{SH}}^{\mathrm{eff}}$ = 1.3.

3. Angle-resolved second harmonic measurement for sample B

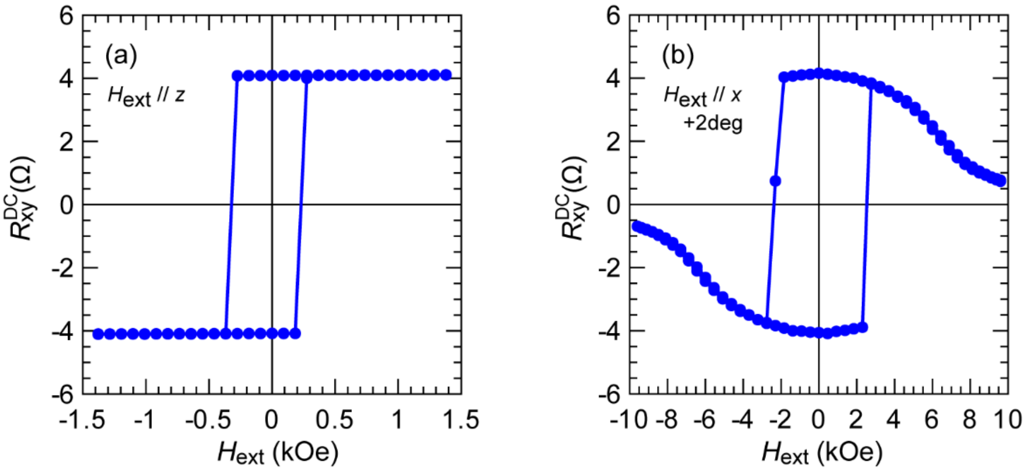

Figure S3. (b) DC anomalous Hall resistance for sample B (σ_{YPtBi} = 1.2×10^5 Ω^{-1}m^{-1} and t_{Co}=0.5 nm) measured with H_{ext} applied along (a) the *z*-direction and (b) the *x*+2°-direction.

Figures S3(a) and S3(b) show the DC anomalous Hall resistance for sample B measured at room temperature with H_{ext} applied along the *z*-direction and *x*+2°-direction. From Fig. S3(b), we found that H_{K}^{eff} of sample B is as large as 7.4 kOe. This makes it difficult to estimate the effective spin Hall angle by the high-field second harmonic technique in this sample. Instead, we performed the angle-resolved second harmonic measurement for this sample.

We derive the formulas for the angle-resolved second harmonic measurement. First, we consider the situation that no current is applied. Then, the magnetic energy E can be written by,

$$\frac{E}{M_S} = -\frac{1}{2}\boldsymbol{m}\cdot\boldsymbol{H}_{\mathbf{k}}^{\mathbf{eff}} - \boldsymbol{m}\cdot\boldsymbol{H}_{\mathbf{ext}}, \tag{S2}$$

where, $\boldsymbol{m}$ is a magnetization unit vector and $\boldsymbol{H}_{\mathbf{k}}^{\mathbf{eff}}$ is given by,

$$\boldsymbol{H}_{\mathbf{k}}^{\mathbf{eff}} = H_{k}^{eff} m_z \hat{\boldsymbol{z}} = \left(\frac{2K_U}{M_S} - 4\pi M_S\right) m_z \hat{\boldsymbol{z}}, \tag{S3}$$

where K_U is a unidirectional magnetic anisotropy energy. By using the spherical coordination system for the magnetization unit vector as shown in Fig. S4, (S2) can be written as,

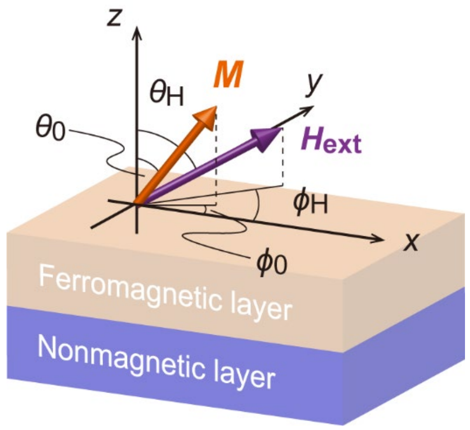

Figure S4. Coordination system and schematic illustration for angle resolved second harmonic measurement.

$$\frac{E}{M_\mathrm{S}} = -\frac{1}{2}H_\mathrm{k}^\mathrm{eff}\cos^2\theta - [(H_\mathrm{ext}^\mathrm{x}\cos\phi + H_\mathrm{ext}^\mathrm{y}\sin\phi)\sin\theta + H_\mathrm{ext}^\mathrm{z}\cos\theta]. \quad \text{(S4)}$$

When the magnetization reaches an equilibrium direction (θ_0, ϕ_0), differentials of equation (S4) by θ and ϕ should be zero at (θ_0, ϕ_0), thus

$$\frac{1}{M_\mathrm{S}}\frac{\partial E}{\partial\theta}(\theta_0, \phi_0) = \frac{1}{2}H_\mathrm{k}^\mathrm{eff}\sin 2\theta_0 - [(H_\mathrm{ext}^\mathrm{x}\cos\phi_0 + H_\mathrm{ext}^\mathrm{y}\sin\phi_0)\cos\theta_0 - H_\mathrm{ext}^\mathrm{z}\sin\theta_0] = 0, \quad \text{(S5)}$$

$$\frac{1}{M_\mathrm{S}}\frac{\partial E}{\partial\phi}(\theta_0, \phi_0) = (H_\mathrm{ext}^\mathrm{x}\sin\phi_0 - H_\mathrm{ext}^\mathrm{y}\cos\phi_0)\sin\theta_0 = 0. \quad \text{(S6)}$$

If a current is applied, the equilibrium direction of magnetization will slightly change due to a spin induced effective filed $\Delta\boldsymbol{H}_\mathbf{SOT}$. This change $\Delta\theta$ and $\Delta\phi$ can be written by,

$$\Delta\theta = \frac{\partial\theta_0}{\partial H_\mathrm{SOT}^\mathrm{x}}\Delta H_\mathrm{SOT}^\mathrm{x} + \frac{\partial\theta_0}{\partial H_\mathrm{SOT}^\mathrm{y}}\Delta H_\mathrm{SOT}^{y} + \frac{\partial\theta_0}{\partial H_\mathrm{SOT}^{z}}\Delta H_\mathrm{SOT}^{z}, \quad \text{(S7)}$$

$$\Delta\phi = \frac{\partial\phi_0}{\partial H_\mathrm{SOT}^\mathrm{x}}\Delta H_\mathrm{SOT}^\mathrm{x} + \frac{\partial\phi_0}{\partial H_\mathrm{SOT}^\mathrm{y}}\Delta H_\mathrm{SOT}^{y} + \frac{\partial\phi_0}{\partial H_\mathrm{SOT}^{z}}\Delta H_\mathrm{SOT}^{z}. \quad \text{(S8)}$$

Here, we approximate $\partial\theta_0/\partial H_\mathrm{SOT}^\mathrm{i}$ and $\partial\phi_0/\partial H_\mathrm{SOT}^\mathrm{i}$ by $\partial\theta_0/\partial H_\mathrm{ext}^\mathrm{i}$ and $\partial\phi_0/\partial H_\mathrm{ext}^\mathrm{i}$ (i = x, y, z), respectively. $\partial\theta_0/\partial H_\mathrm{ext}^\mathrm{i}$ and $\partial\phi_0/\partial H_\mathrm{ext}^\mathrm{i}$ can be obtained from equations (S5) and (S6) with differentials by $H_\mathrm{ext}^\mathrm{i}$,

$$\frac{1}{M_\mathrm{S}}\frac{\partial^2 E}{\partial H_\mathrm{ext}^\mathrm{i}\partial\theta}(\theta_0, \phi_0) = [H_\mathrm{k}^\mathrm{eff}\cos 2\theta_0 + (H_\mathrm{ext}^\mathrm{x}\cos\phi_0 + H_\mathrm{ext}^\mathrm{y}\sin\phi_0)\sin\theta_0 + H_\mathrm{ext}^\mathrm{z}\cos\theta_0]\frac{\partial\theta_0}{\partial H_\mathrm{ext}^\mathrm{i}}$$
$$+(H_\mathrm{ext}^\mathrm{x}\sin\phi_0 - H_\mathrm{ext}^\mathrm{y}\cos\phi_0)\cos\theta_0\frac{\partial\phi_0}{\partial H_\mathrm{ext}^\mathrm{i}} - f_\mathrm{i} = 0, \quad \text{(S9)}$$

$$\frac{1}{M_\mathrm{S}}\frac{\partial^2 E}{\partial H_\mathrm{ext}^\mathrm{i}\partial\phi}(\theta_0,\phi_0) = (H_\mathrm{ext}^\mathrm{x}\sin\phi_0 - H_\mathrm{ext}^\mathrm{y}\cos\phi_0)\cos\theta_0\frac{\partial\theta_0}{\partial H_\mathrm{ext}^\mathrm{i}}$$
$$+(H_\mathrm{ext}^\mathrm{x}\cos\phi_0 + H_\mathrm{ext}^\mathrm{y}\sin\phi_0)\sin\theta_0\frac{\partial\phi_0}{\partial H_\mathrm{ext}^\mathrm{i}} - g_i = 0, \quad \text{(S10)}$$

where, f_i and g_i are given by

$$f_\mathrm{i} = (\cos\theta_0\cos\phi_\mathrm{H}\,,\quad \cos\theta_0\sin\phi_\mathrm{H},\quad -\sin\theta_0), \quad \text{(S11)}$$
$$g_i = (-\sin\theta_0\sin\phi_{\mathrm{H},}\quad \sin\theta_0\cos\phi_\mathrm{H},\quad 0). \quad \text{(S12)}$$

For simplicity, we assume that an in-plane component of magnetization is determined by the direction of H_ext, which is reasonable except for samples with a strong in-plane easy axis such as nano-wires. When H_ext is applied in the *xz*-plane under this approximation, equations (S9) and (S10) are written as,

$$[H_\mathrm{k}^\mathrm{eff}\cos 2\theta_0 + |H_\mathrm{ext}|\cos(\theta_\mathrm{H}-\theta_0)]\frac{\partial\theta_0}{\partial H_\mathrm{ext}^\mathrm{i}} = f_\mathrm{i}, \quad \text{(S13)}$$

$$|H_\mathrm{ext}|\sin\theta_\mathrm{H}\sin\theta_0\frac{\partial\phi_0}{\partial H_\mathrm{ext}^\mathrm{i}} = g_i. \quad \text{(S14)}$$

$$f_\mathrm{i} = (\cos\theta_0\,,\quad 0,\quad -\sin\theta_0), \quad \text{(S15)}$$
$$g_i = (0,\quad \sin\theta_0,\quad 0). \quad \text{(S16)}$$

where, θ_H is the polar angle of H_ext as shown in Fig. S4. Therefore, $\Delta\theta$ and $\Delta\phi$ are given by,

$$\Delta\theta = \frac{\Delta H_\mathrm{SOT}^\mathrm{x}\cos\theta_0 - \Delta H_\mathrm{SOT}^\mathrm{z}\sin\theta_0}{H_\mathrm{k}^\mathrm{eff}\cos 2\theta_0 + |H_\mathrm{ext}|\cos(\theta_\mathrm{H}-\theta_0)}, \quad \text{(S17)}$$

$$\Delta\phi = \frac{\Delta H_\mathrm{SOT}^\mathrm{y}}{|H_\mathrm{ext}|\sin\theta_\mathrm{H}}. \quad \text{(S18)}$$

Here, when a current is applied along the *x*-direction, $\Delta\boldsymbol{H}_\mathbf{SOT}$ is given by,

$$\Delta\boldsymbol{H}_\mathbf{SOT} = (H_\mathrm{DL}\cos\theta_0\,,\quad -H_\mathrm{FL+OF},\quad -H_\mathrm{DL}\sin\theta_0\cos\phi_H)$$
$$= (H_\mathrm{DL}\cos\theta_0\,,\quad -H_\mathrm{FL+OF},\quad -H_\mathrm{DL}\sin\theta_0), \quad \text{(S19)}$$

and thus, we finally obtain $\Delta\theta$ and $\Delta\phi$ as,

$$\Delta\theta = \frac{H_\mathrm{DL}}{H_\mathrm{k}^\mathrm{eff}\cos 2\theta_0 + |H_\mathrm{ext}|\cos(\theta_\mathrm{H}-\theta_0)}, \quad \text{(S20)}$$

$$\Delta\phi = -\frac{H_\mathrm{FL+OF}}{|H_\mathrm{ext}|\sin\theta_\mathrm{H}}. \quad \text{(S21)}$$

When an AC current is applied, $\Delta\theta$ and $\Delta\phi$ also change with the same frequency with the current. Then, the effects of $\Delta\theta$ and $\Delta\phi$ appear in a second harmonic Hall resistance because the anomalous Hall and planar Hall resistances can be expressed as follows by using Taylor expansion,

$$R_\mathrm{AHE}\cos(\theta_0+\Delta\theta) \sim R_\mathrm{AHE}\cos\theta_0 - R_\mathrm{AHE}\sin\theta_0\,\Delta\theta, \quad \text{(S22)}$$

$$R_{\mathrm{PHE}}\sin^2(\theta_0+\Delta\theta)\sin 2(\phi_0+\Delta\phi)\sim R_{\mathrm{PHE}}\sin^2\theta_0\sin 2\phi_0$$
$$+R_{\mathrm{PHE}}\sin 2\theta_0\sin 2\phi_0\,\Delta\theta+2R_{\mathrm{PHE}}\sin^2\theta_0\cos 2\phi_0\,\Delta\phi. \qquad \text{(S23)}$$

By considering the ordinary Nernst effect (ONE), the anomalous Nernst effect (ANE) and the spin Seebeck effect (SSE) induced by a temperature gradient along the *z*-direction, the second harmonic Hall resistance at ϕ_0= 0 is given by,

$$R_{\mathrm{xy}}^{2\omega}=\frac{R_{\mathrm{AHE}}}{2}\sin\theta_0\,\Delta\theta-R_{\mathrm{PHE}}\sin^2\theta_0\,\Delta\phi+\alpha_{\mathrm{ONE}}\sin\theta_{\mathrm{H}}\,|H_{\mathrm{ext}}|+R_{\mathrm{ANE+SSE}}\sin\theta_0\,, \qquad \text{(S24)}$$

where, α_{ONE} is a coefficient originated from the ONE and $R_{\mathrm{ANE+SSE}}$ is a second harmonic Hall resistance originated from the ANE and SSE. By substituting equation (S20) and (S21) into equation (S24), we obtain

$$R_{\mathrm{xy}}^{2\omega}=\frac{R_{\mathrm{AHE}}}{2}\sin\theta_0\frac{H_{\mathrm{DL}}}{H_{\mathrm{K}}^{\mathrm{eff}}\cos 2\theta_0+|H_{\mathrm{ext}}|\cos(\theta_{\mathrm{H}}-\theta_0)}+R_{\mathrm{PHE}}\sin^2\theta_0\frac{H_{\mathrm{FL+OF}}}{|H_{\mathrm{ext}}|\sin\theta_{\mathrm{H}}}$$
$$+\alpha_{\mathrm{ONE}}\sin\theta_{\mathrm{H}}\,|H_{\mathrm{ext}}|+R_{\mathrm{ANE+SSE}}\sin\theta_0\,. \qquad \text{(S25)}$$

Here, we can obtain the following equation from equation (S5) for small θ_0 and θ_{H},

$$\theta_0=\frac{|H_{\mathrm{ext}}|}{|H_{\mathrm{ext}}|+H_{\mathrm{k}}^{\mathrm{eff}}}\theta_{\mathrm{H}}. \qquad \text{(S26)}$$

Therefore, equation (S25) with small θ_0 and θ_{H} also can be written as,

$$R_{\mathrm{xy}}^{2\omega}=\pm\left(\frac{R_{\mathrm{AHE}}}{2}H_{\mathrm{DL}}+R_{\mathrm{PHE}}H_{\mathrm{FL+OF}}\right)\frac{|H_{\mathrm{ext}}|\theta_{\mathrm{H}}}{(|H_{\mathrm{ext}}|+H_{\mathrm{k}}^{\mathrm{eff}})^2}\pm\alpha_{\mathrm{ONE}}\theta_{\mathrm{H}}|H_{\mathrm{ext}}|$$
$$\pm R_{\mathrm{ANE+SSE}}\frac{|H_{\mathrm{ext}}|\theta_{\mathrm{H}}}{|H_{\mathrm{ext}}|+H_{\mathrm{k}}^{\mathrm{eff}}}, \qquad \text{(S27)}$$

where, the sign $\pm$ corresponds to the *z*-direction of H_{ext} or $\boldsymbol{m}$. Finally, by calculating the differential of (S27) by θ_{H}, we obtain

$$\frac{1}{|H_{\mathrm{ext}}|}\frac{dR_{\mathrm{xy}}^{2\omega}}{d\theta_{\mathrm{H}}}=\pm\left(\frac{R_{\mathrm{AHE}}}{2}H_{\mathrm{DL}}+R_{\mathrm{PHE}}H_{\mathrm{FL+OF}}\right)(|H_{\mathrm{ext}}|+H_{\mathrm{k}}^{\mathrm{eff}})^{-2}$$
$$\pm R_{\mathrm{ANE+SSE}}(|H_{\mathrm{ext}}|+H_{\mathrm{k}}^{\mathrm{eff}})^{-1}\pm\alpha_{\mathrm{ONE}}. \qquad \text{(S28)}$$

From (S28), we can see that the SOT contribution has a $(|H_{\mathrm{ext}}|+H_{\mathrm{k}}^{\mathrm{eff}})^{-2}$ dependence, the ANE and SSE contribution have a $(|H_{\mathrm{ext}}|+H_{\mathrm{k}}^{\mathrm{eff}})^{-1}$ dependence, while the ONE has a constant contribution to $\frac{1}{|H_{\mathrm{ext}}|}\frac{dR_{\mathrm{xy}}^{2\omega}}{d\theta_{\mathrm{H}}}$. Thus, by measuring $\frac{dR_{\mathrm{xy}}^{2\omega}}{d\theta_{\mathrm{H}}}$ as a function of H_{ext} at various small θ_{H} and fitting to (S28), we can separate the SOT contribution from that of the thermal effects.

Figure S5(a) show a representative $R_{\mathrm{xy}}^{2\omega}$ - θ_{H} data measured at H_{ext} = 5.1 kOe and an AC current of 3.4 mA. The slopes correspond to $dR_{\mathrm{xy}}^{2\omega}/d\theta_{\mathrm{H}}$ in equation (S28). Figure S5(b) shows $|H_{\mathrm{ext}}|^{-1}dR_{\mathrm{xy}}^{2\omega}/d\theta_{\mathrm{H}}$ as a function of $(|H_{\mathrm{ext}}|+H_{\mathrm{k}}^{\mathrm{eff}})^{-1}$ and the

corresponding fitting at bias currents of 3.4 mA, where the dots and solid curves are the experimental data and fitting results given by equation (S28), respectively. From the coefficient of $(|H_{\text{ext}}|+H_{\text{k}}^{\text{eff}})^{-2}$ term (ignoring the $R_{\text{PHE}}H_{\text{FL+OF}}$ contribution), M_S = 729 emu/cc and t_{CoPt} = 1.5 nm, we obtained $\theta_{\text{SH}}^{\text{eff}}$ = 0.9±0.2 for sample B.

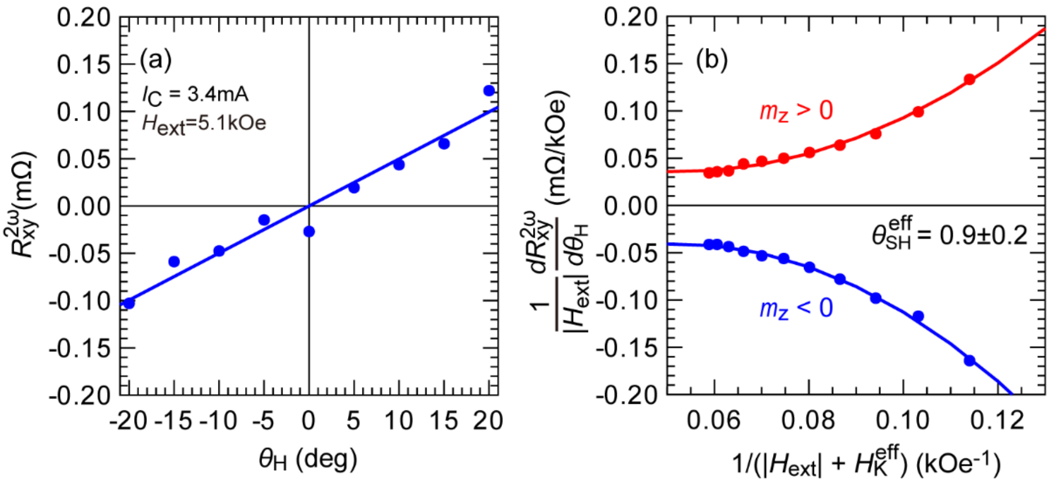

Figure S5. (a) Second harmonic Hall resistance of sample B as a function of θ_H measured with H_{ext} = 5.1 kOe and an AC current of 3.4 mA, where dots are experimental data and the solid line shows the gradient $dR_{xy}^{2\omega}/d\theta_H$. (b) $|H_{\text{ext}}|^{-1}dR_{xy}^{2\omega}/d\theta_H$ (dots) as a function of $(|H_{\text{ext}}|+H_{\text{k}}^{\text{eff}})^{-1}$ and the corresponding fitting curves using equation (S28).

4. High-field second harmonic measurement of sample C

Since the sample C has the highest surface quality, $H_{\text{k}}^{\text{eff}}$ of Pt/Co/Pt could be even larger than that of sample B. To reduce $H_{\text{k}}^{\text{eff}}$, we increased the Co thickness from 0.5 nm to 0.8 nm in sample C. This allows us to perform the high-field second harmonic measurement on sample C, which is quicker and more simple than the angle-resolved second harmonic technique.

Figures S6(a) and S6(b) show the DC anomalous Hall resistance for sample C measured with H_{ext} applied along the z-direction and x+2°-direction. We obtain $H_{\text{k}}^{\text{eff}}$ = 6.1 kOe from Fig. S6(b). Figure S6(c) shows the high-field second harmonics data and the corresponding fitting for sample C at bias currents of 1.0 to 3.4 mA, where the dots and solid curves are the experimental data and fitting using equation (2) in the main text, respectively. Figure S6(d) shows the relationship between the extracted values of H_{DL} and J_{YPtBi} for sample C. From the slope H_{DL} / J_{YPtBi}, M_S = 837 emu/cc and t_{CoPt} = 1.8 nm, we obtained $\theta_{\text{SH}}^{\text{eff}}$ = 0.64 in sample C.

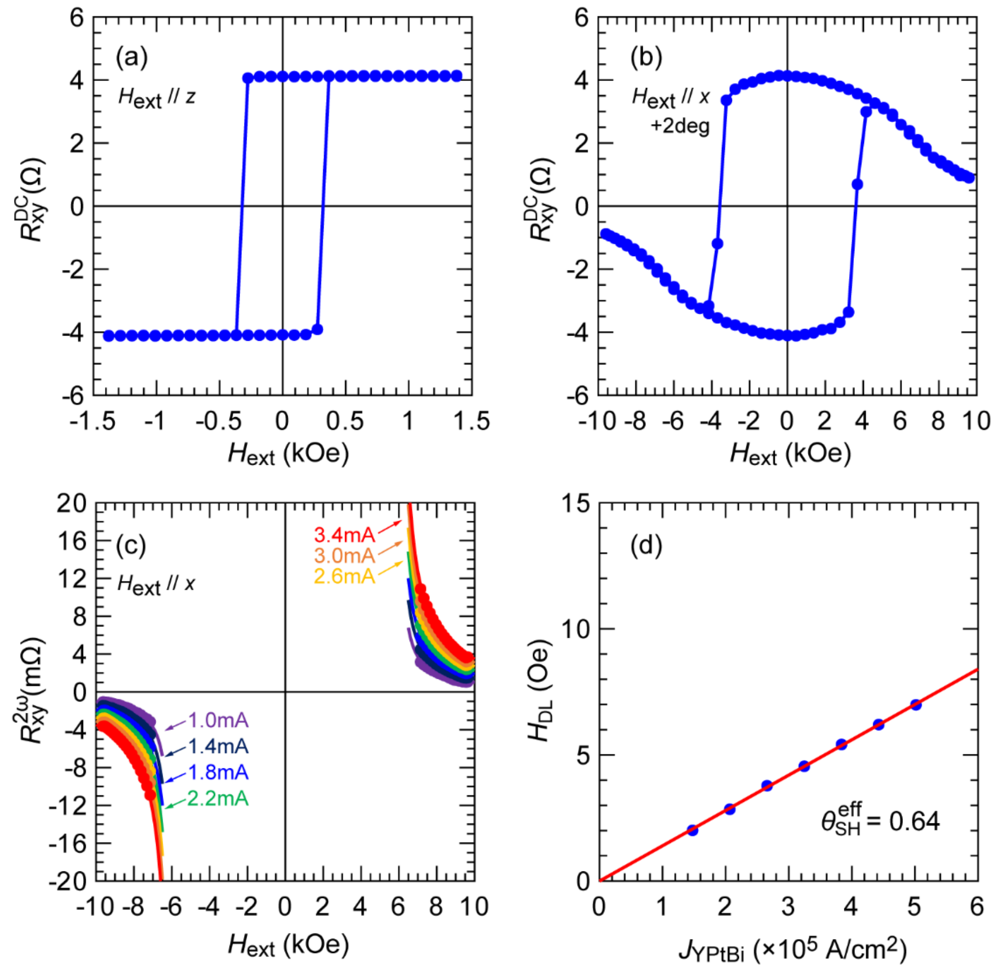

Figure S6. (a), (b) DC anomalous Hall resistance for sample C (σ_{YPtBi} = 1.5×10^5 $\Omega^{-1}m^{-1}$ and t_{Co}=0.8 nm) measured with H_{ext} applied along the *z*-direction and the *x*+2°-direction, respectively. (c) Second harmonic Hall resistance of sample C measured with H_{ext} applied along the *x*-direction and an AC current ranging from 1.0 to 3.4 mA, where dots are experimental data and solid curves show fitting results given by equation (2) in the main text. (d) Antidamping-like field H_{DL} as a function of the current density in the YPtBi layer.

5. High-field second harmonic measurement of sample D

Figures S7(a) and S7(b) show the DC anomalous Hall resistance for sample D measured at room temperature with H_{ext} applied along the *z*-direction and *x*+5°-direction. We obtained H_k^{eff} = 1.2 kOe from Fig. S7(b). Figure S7(c) shows the high-field second harmonics data and the corresponding fitting for sample D at bias currents of 2.0 to 5.0 mA, where the dots and solid curves are the experimental data and fitting using equation

(2) in the main text, respectively. Figure S7(d) shows the relationship between the extracted values of H_{DL} and J_{YPtBi} for sample D. From the slope H_{DL} / J_{YPtBi}, M_S = 412 emu/cc and t_{CoPt} = 2.1 nm, θ_{SH}^{eff} of 0.12 was obtained in sample D.

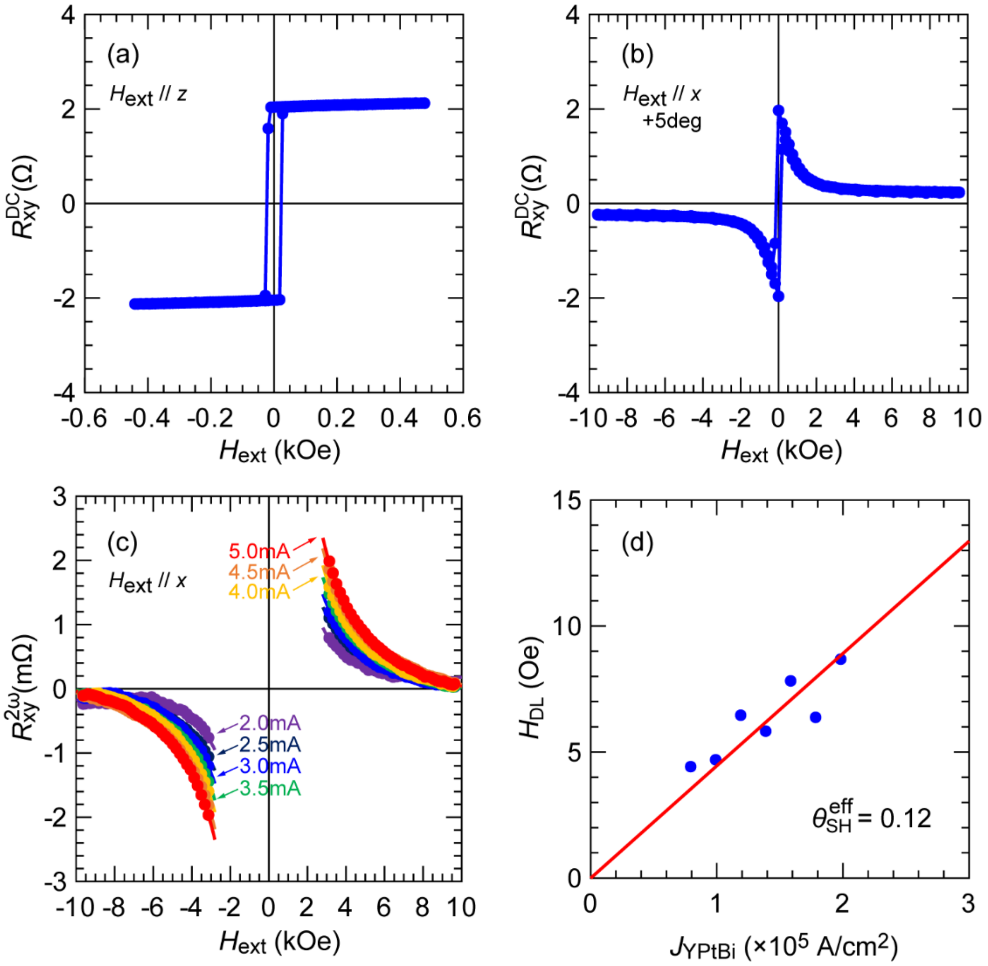

Figure S7. (a), (b) DC anomalous Hall resistance for sample D (σ_{YPtBi} = 2.0×10^5 Ω^{-1}m^{-1} and t_{Co}=0.5 nm) measured with H_{ext} applied along the z-direction and the x+5°-direction, respectively. (c) Second harmonic Hall resistance of sample D measured with H_{ext} applied along the x-direction and an AC current ranging from 2.0 to 5.0 mA, where dots are experimental data and solid curves show fitting results given by equation (2) in the main text. (d) Antidamping-like field H_{DL} as a function of the current density in the YPtBi layer.

6. High-field second harmonic measurement of sample E

Figures S8(a) and S8(b) show the DC anomalous Hall resistance for sample E measured at room temperature with H_{ext} applied along the z-direction and x+5°-direction.

We obtained H_k^{eff} = 3.4 kOe from Fig. S8(b). Figure S8(c) shows the high-field second harmonics data and the corresponding fitting for sample E at bias currents of 1.0 to 3.0 mA, where the dots and solid curves are the experimental data and fitting using equation (2) in the main text, respectively. Figure S8(d) shows the relationship between the extracted values of H_{DL} and J_{YPtBi} for sample E. From the slope H_{DL} / J_{YPtBi}, M_S = 435 emu/cc and t_{CoPt} = 2.1 nm, θ_{SH}^{eff} of 1.6 was obtained in sample E.

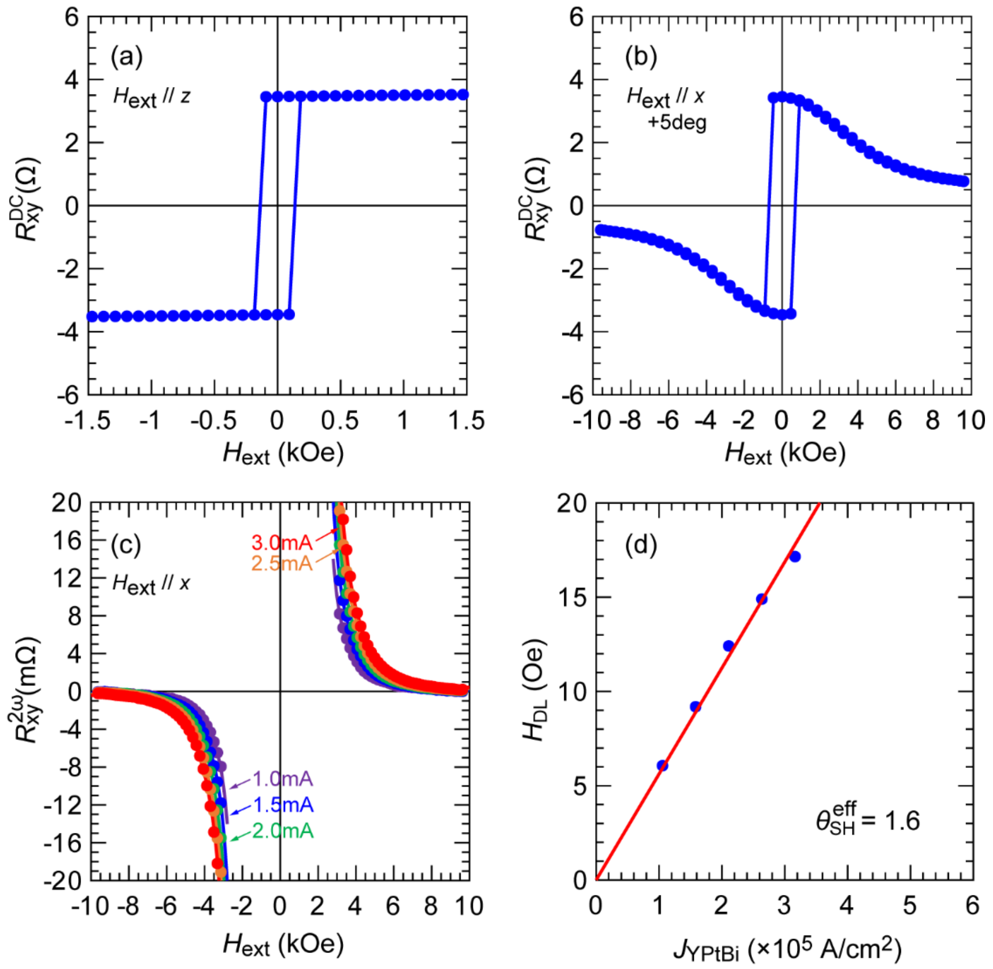

Figure S8. (a), (b) DC anomalous Hall resistance for sample E (σ_{YPtBi} = 0.39×10^5 Ω^{-1}m^{-1} and t_{Co}=0.5 nm) measured with H_{ext} applied along the z-direction and the x+5°-direction, respectively. (c) Second harmonic Hall resistance of sample E measured with H_{ext} applied along the x-direction and an AC current ranging from 1.0 to 3.0 mA, where dots are experimental data and solid curves show fitting results given by equation (2) in the main text. (d) Antidamping-like field H_{DL} as a function of the current density in the YPtBi layer.
